# Three-Dimensional Quantification of Cellular Traction Forces and Mechanosensing of Thin Substrata by Fourier Traction Force Microscopy

**DOI:** 10.1371/journal.pone.0069850

**Published:** 2013-09-04

**Authors:** Juan C. del Álamo, Ruedi Meili, Begoña Álvarez-González, Baldomero Alonso-Latorre, Effie Bastounis, Richard Firtel, Juan C. Lasheras

**Affiliations:** 1 Mechanical and Aerospace Engineering Department, University of California San Diego, La Jolla, California, United States of America; 2 Institute for Engineering in Medicine, University of California San Diego, La Jolla, California, United States of America; 3 Division of Cell and Developmental Biology, University of California San Diego, La Jolla, California, United States of America; 4 Bioengineering Department, University of California San Diego, La Jolla, California, United States of America; University of Cambridge, United Kingdom

## Abstract

We introduce a novel three-dimensional (3D) traction force microscopy (TFM) method motivated by the recent discovery that cells adhering on plane surfaces exert both in-plane and out-of-plane traction stresses. We measure the 3D deformation of the substratum on a thin layer near its surface, and input this information into an exact analytical solution of the elastic equilibrium equation. These operations are performed in the Fourier domain with high computational efficiency, allowing to obtain the 3D traction stresses from raw microscopy images virtually in real time. We also characterize the error of previous two-dimensional (2D) TFM methods that neglect the out-of-plane component of the traction stresses. This analysis reveals that, under certain combinations of experimental parameters (cell size, substratums' thickness and Poisson's ratio), the accuracy of 2D TFM methods is minimally affected by neglecting the out-of-plane component of the traction stresses. Finally, we consider the cell's mechanosensing of substratum thickness by 3D traction stresses, finding that, when cells adhere on thin substrata, their out-of-plane traction stresses can reach four times deeper into the substratum than their in-plane traction stresses. It is also found that the substratum stiffness sensed by applying out-of-plane traction stresses may be up to 10 times larger than the stiffness sensed by applying in-plane traction stresses.

## Introduction

Adherent cells exert mechanical forces on the extracellular matrix (ECM) to regulate adhesions, propel their migration [1] and to sense the ECM stiffness by a process generally referred as mechanosensing [2], [3]. In our organism, cells are often embedded in three-dimensional (3D) ECMs, which they deform in all spatial directions by generating three-dimensional forces in order to migrate [4]. Even cells that form stable two-dimensional (2D) monolayers, such as vascular endothelial cells, are known to exert three-dimensional traction forces [5] both in isolation and confluency [6]. The ability of cells to apply three-dimensional forces on two-dimensional layers is also important for the extravasation of leukocytes during the immune response [7] and for cancer cell invasion [8], [9].

The past few years have witnessed the development of several 2D traction force microscopy (TFM) methods, which allow investigators to measure only the in-plane (tangential) components of the traction stresses generated by cells adhering to plane substrata [10]–[15]. More recently, 3D TFM methods have been introduced allowing for the determination of the out-of-plane (normal) component of the traction stresses [5], [16]. However, these 3D TFM methods are based on numerical calculations performed on large volumetric grids, which can limit their ability to deal with large experimental sets containing many samples. Furthermore, some of these techniques [16] rely on the acquisition of thick *z*-stacks containing a large number of confocal images, which not only subjects the cells to high levels of laser radiation, but also constrains the temporal resolution of the measurements.

The realization that cells generate out-of-plane traction stresses even when they adhere to plane surfaces [5] has prompted an increasing demand for the characterization of the error that the widely used 2D TFM methods may have incurred by neglecting these stresses. This would require to quantify the error of 2D TFM as a function of experimental parameters such as cell size, cell shape, substratum thickness and substratum Poisson ratio (see 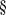 3.2 for details). Because existing 3D TFM methods are not well suited for parametric studies, the error of 2D TFM has not been characterized yet.

The effect of the normal traction stresses on ECM mechanosensing is another aspect related to 3D traction stresses that remains unexplored. Since normal stresses deform the substratum axially while tangential ones shear it, and these two types of deformation are different from each other, it is reasonable to question if cells can sense different ECM mechanical properties by imparting tangential or normal stresses. Thick isotropic gels have their shear elastic modulus proportional to their axial Young's modulus and, thus, they exhibit similar stiffness under both tangential and normal traction stresses [17]. However, the same cannot be expected to hold for thin substrata, where the contact with a rigid surface (a glass coverslip *in vitro* or bone tissue *in vivo*) breaks the macroscopic isotropy condition. of the system as a whole, even if the microscopic material properties of the gel remain isotropic. The elastic response to tangential traction stresses in thin substrata has been previously studied using 2D TFM approaches [18], [19] but we still lack information about the response to normal traction stresses. Harnessing the different values of the elastic modulus sensed by the cell by tangential and normal traction stresses could provide some control over the cellular response to an ECM with given mechanical properties.

The aim of this paper is to address these open questions and to improve existing 3D TFM approaches by introducing a new traction cytometry method based on an exact analytical three-dimensional solution to the elastic equilibrium equation for a substratum of finite thickness. In this method, it is sufficient to measure the 3D deformation of the substratum on a thin layer near its surface. Therefore, the cells are exposed to lower levels of laser radiation and a higher temporal resolution is obtained. The analytical solution derived in this study is a general explicit formula that can be recycled for different sets of deformation measurements, resulting in an improved computational efficiency compared to existing 3D TFM methods. The new methodology is described in section and its performance is illustrated in section 3.1 for chemotaxing *Dictyostelium* cells. Section 3.2 compares the results of 2D and 3D TFM methods for a synthetic 3D deformation field that simulates the deformation patterns measured in the experiments. Specifically, section 3.2.1 defines the error of 2D TFM methods and analyzes its dependence on the experimental parameters in order to obtain the combinations of these parameters that minimize the error. A spectral comparison of 2D and 3D TFM methods that does not presume any synthetic shape for the deformation field is given in section 3.2.4. This spectral comparison is extended in section 3.3 to study mechanosensing of substratum stiffness and thickness by 3D traction stresses.

## Materials, Methods & Analysis

### 2.1 *Dictyostelium* Cell Culture

*Dictyostelium* cells were grown under axenic conditions in HL5 growth medium in tissue culture plates as described in [20]. We used wild type (*WT*) cells (Ax3). Aggregation competent cells were prepared by pulsing a 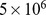 cells/ml suspension in Na/K phosphate buffer (9.6 mM KH_2_PO_4_, 2.4 mM Na_2_HPO_4_, pH 6.3) with cAMP to a concentration of 30 nM every 6 min for 6 h. Cells were seeded onto the functionalized polyacrylamide substratum and allowed to adhere. A drawn glass capillary mounted on a micromanipulator served as the source of chemoattractant (150 µ M cAMP in an Eppendorf femtotip, Eppendorf, Germany).

### 2.2 Polyacrylamide Gel Preparation

12–mm diameter and 50–100 µm thick polyacrylamide gels were fabricated on 22–mm square #1 glass coverslips using essentially the procedure described by Beningo *et al.* [21]. To improve the signal to noise ratio of the *z*-stacks, the polyacrylamide gel was fabricated as two sequential layers with the first bottom one containing no beads and the second top one containing 0.03% carboxylate modified yellow latex beads with 0.1 µm diameter (Fluospheres, Invitrogen, Carlsbad CA). The two layers were verified to adhere well to each other by performing supporting experiments using gels where the beads were contained in the bottom layer. The square coverslips with the two layered gels were then mounted in Petri dishes with a circular opening in the bottom using silicon grease (Dow Corning, Midland, Michigan). We crosslinked collagen I to the surface using Sulfo-SANPAH (Thermo Sci, Rockford, Il) (1 mM in HEPES buffer (pH 8.5). After UV activation and washing thoroughly, 0.25 mg/ml collagen protein in HEPES were added and the gels were incubated overnight at room temperature. After washing the gels were stored with buffer (9.6 mM KH2PO4, 2.4 mM Na2HPO4, pH 6.3, same buffer used in the experiments) and antibiotic (40 µM Ampicillin) for use within a week. Substratum thickness was measured by locating the top and bottom planes of the gel and subtracting their *z* positions. The top plane was found by maximizing the number of in-focus pixels of cell outlines as described by del Álamo *et al.* [13] The bottom plane was found in a similar manner by focusing on streaky patterns left on the surface of the glass coverslip during treatment for gel attachment.

### 2.3 Microscopy

**Figure 1:**
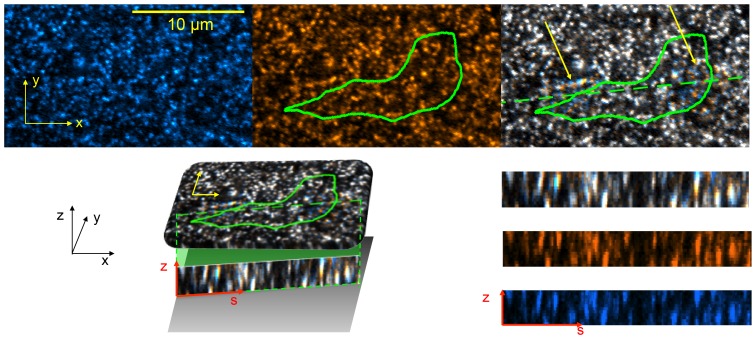
Example of fluorescence confocal image used to determine substratum deformation. The three panels at the top of the figure (*a–c*) show the first horizontal slice of a 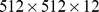
–pixel 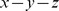 stack, which is focused at the free surface of the substratum (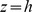). *(* ***a*** *)*, Tracer beads fluorescence in undeformed conditions used as reference for traction force microscopy. The scale bar is 10 microns long. The axes indicate the reference system for both the substratum deformation and the traction stresses. *(* ***b*** *)*, Tracer beads fluorescence when the substratum is deformed by a migrating cell, whose outline is indicated by the green contour. *(* ***c*** *)*, Image obtained by merging the fluorescence from tracer beads in undeformed (*a*) and deformed (*b*) conditions, which reveals the deformation of the substratum. White speckles indicate perfect match between undeformed and deformed conditions and thus zero local deformation. Blue and orange speckles indicate mismatch between undeformed and deformed conditions and thus non-zero local deformation. Regions of locally large deformation are indicated with yellow arrows. The dashed green line indicates the location of the vertical section shown in panels *d–g*. *(* ***d*** *)*, Three-dimensional illustration of the relative positions of the horizontal plane in panel (*x*–*y*, yellow axes) and the vertical plane in panel (*s*–*z*, red axes). The black axes indicate the three-dimensional reference system used to express both the substratum deformation and the traction stresses. The three panels at the bottom right corner of the figure (*e–g*) show vertical slices of the same 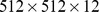
–stack passing through the dashed green line in panel at 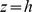. *(* ***e*** *)*, Image obtained by merging the fluorescence from tracer beads in undeformed (*f*) and deformed (*g*) conditions. Speckle patterns with orange top and blue bottom are found in locations where the cell is pulling up on the substratum. Conversely, speckle patterns with blue top and orange bottom are found the when cell is pushing down on the substratum. *(* ***f*** *)*, Tracer beads fluorescence in deformed condition. *(* ***g*** *)*, Tracer beads fluorescence in underformed condition.

The 3D deformation of the polyacrylamide substratum was determined from the displacements of embedded 0.1–µm fluorescent beads as described in section 2.5 (see Figure 1). Time-lapse sequences of *z*-stacks of fluorescence images were acquired using a Marinas Workstation, 3I, Denver CO. 3I Marianas spinning disk confocal system. It consist of a Zeiss Observer Z1 body with a 63× oil immersion lens, a Yokogawa CSU-X1 spinning disk head and a Roper Quant EM 512 SC camera for image capture. The pinholes have a diameter of 50 µm. The measured full-width half maximum of the point spread function of the system was measured to be 1.4 µm. Each *z*-stack contained 8–12 images separated a vertical distance 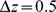 µm. The images were collected using an 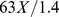 oil lens through a spinning disk confocal head. The *xy* position and the shape of the cells was recorded with an additional single differential interference contrast (DIC) transmitted light image for each time point.

### 2.4 Identification of cell contours

Cell outlines were determined from the DIC images of the free surface of the substratum captured as described in 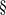 2.3. Image processing was performed with MATLAB (Mathworks Inc, Natick, MA) as described in previous works by our group [13], [22], [23]. Static imperfections were removed from individual images using the average of the image series. A threshold was applied to the resulting images to extract the most intense features, which were refined using two consecutive image dilations and erosions with structuring elements of increasing size. The sets of connected pixels were detected and their holes were filled.

### 2.5 Measurement of Three-dimensional Substrate Deformation

The deformation of the substratum was measured in three dimensions by cross-correlating each instantaneous fluorescence *z*-stack obtained with the confocal microscope (see 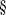 2.3) with a reference *z*-stack in which the substratum is not deformed (see Figure 1). In each experiment, the undeformed image was obtained by waiting for the cell to move out of the field of view, which takes approximately 10 minutes as *Dictyostelium* cells are highly motile. The comparison between the deformed and undeformed (reference) conditions was performed by dividing each instantaneous and reference *z*-stacks into 3D interrogation boxes and optimizing the 3D cross-correlation between each pair of interrogation boxes. Sub-pixel resolution was attained by tri-quadratic polynomial interpolation of the image correlation function. The image correlation codes were validated against the 3D single-particle tracking codes developed by Hur *et al.* [5], showing very good agreement. Figure S2 in file Supporting Information (File S1) shows a representative example of the 3D cross-correlation function between interrogation boxes from the deformed and undeformed images. The signal-to-noise ratio of the measurement is defined as the ratio between the maximum value of the cross-correlation and the second highest local maximum. The measurement floor estimates the lowest deformation that could be accurately measured by our method. It was defined from the standard deviation of the deformation measured far away from the cell, where this deformation should be equal to zero. The horizontal size of the interrogation boxes was chosen to balance resolution, which decreased with box size, measurement floor, which decreased with box size, and signal-to-noise ratio, which increased with box size (see Table 1). We chose the smallest box size that provided a signal-to-noise ratio greater than 2.5 which resulted in a box with 24×24 pixels in the *x* and *y* directions, and led to a Nyquist spatial resolution of 2.1 µ*m*. Further increases in box size led to higher signal to noise ratios but did not decrease the measurement floor appreciably. Typical values of deformations exerted by the cells ranged between 30 and 50 times the measurement floor. In addition to these considerations, phototoxic effects need to be taken into account when choosing the vertical size of the interrogation boxes, 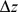, because the level of laser radiation transmitted to the cells increases with the number of slices per *z*-stack. In our experiments, 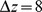 was the minimum box height that allowed for meaningful deformation measurements. Thus, we acquired *z*-stacks having between 8 and 12 slices, which enabled us to record time lapse sequences of stacks with a time resolution of 4 seconds and durations of up to 30 minutes with no apparent phototoxic effects.

**Table 1:** Average signal-to-noise ratio of the three-dimensional displacement measurements obtained by image cross-correlation as a function of the size of the interrogation box (in pixels).

Interrogation Box Size	Nyquist resolution	Signal-to-noise Ratio	Displacement floor	
(Δ*x*×Δ*y*, pixels)	(µm)		*u*–*v* (µm)	*w* (µm)
8×8	0.7	1.8	0.014	0.015
16×16	1.4	2.3	0.009	0.008
24×24	2.1	2.6	0.007	0.006
32×32	2.7	2.9	0.007	0.006
64×64	5.6	3.5	0.007	0.005

Signal-to-noise ratio is defined as the ratio between the maximum value of the cross-correlation and the second local maximum. All data in this table have been obtained for interrogation boxes with vertical size of 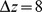 pixels.

### 2.6 Calculation of Three-dimensional Traction Stresses

**Figure 2:**
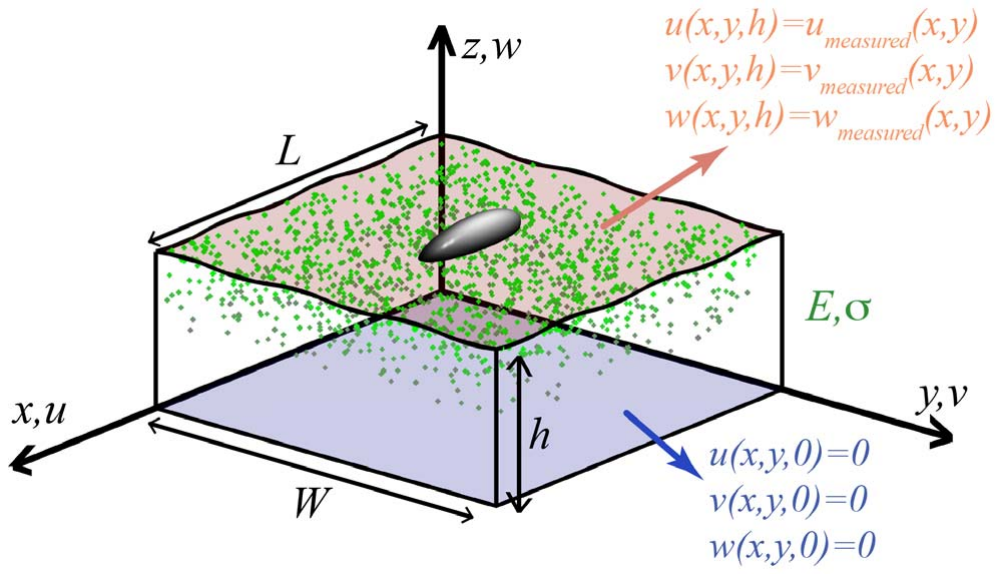
Configuration of the 3D TFM mathematical problem. The input data are the measured three-dimensional deformation caused by the cell on the free surface of the substratum (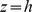, red), and it is assumed that the deformation of the substratum is zero at the bottom surface in contact with the glass coverslip (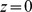, blue). We assume that the substratum has linear, homogeneous, isotropic material properties, with Young modulus *E* and Poisson's ratio *σ*. Fourier series with spatial periods *L* and *W* are used to express the dependence of the variables in the horizontal directions.

We computed the three-dimensional deformation field in the whole substratum by solving the elasticity equation of equilibrium for a linear, homogeneous, isotropic three-dimensional body characterized by its Poisson's ratio, *σ*,
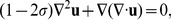
(1)where the inertial stresses have been neglected. This assumption is justified by estimating that the ratio between the inertial and elastic stresses is of order 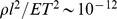, where 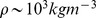 and 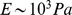 are respectively the density and Young modulus of the substratum, 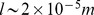 is the length of the cell and 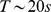 is the characteristic timescale of pseudopod protrusion and retraction [24]. We sought for solutions of this equation that are periodic in both horizontal directions and expressed them using a discrete Fourier series,
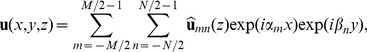
(2)where 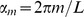, 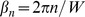 are respectively the wavenumbers in the *x* and *y* directions, while *L* and *W* are respectively and the spatial periods of the domain. The numbers of Fourier coefficients in each direction are determined from the PIV grid of interrogation boxes using the Nyquist criterion, yielding 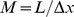 and 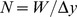. Figure 2 outlines the problem configuration. For the sake of brevity and without loss of generality, we will drop the subindices *m* and *n* of *α* and *β* in what follows (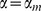 and 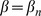).

The boundary conditions to eq. 1 are partially set up by imposing zero displacements at the base of the substratum, i.e. 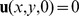 (see Fig. 2), since the glass coverslip is much stiffer than the substratum gel, and the gel is firmly adhered to the coverslip. A general solution to eq. 1 that is compatible with zero displacements at the bottom of the gel was determined by del Álamo *et al.* [13]. More recently, an elegantly simple solution to the 2D problem that only requires computing two scalar functions was found by Trepat *et al.* [15]. However, here we follow the more general formulation by del Álamo *et al.* [13], which we can easily extend to three dimensions and which is also relatively simple. We express the Fourier transform of the deformation vector as
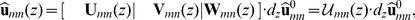
(3)where 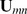, 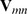, 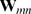 are three fundamental solutions of the problem (given in the Supporting Information, File S1). Thus, 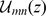 is the resolvant matrix. The vector 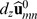 contains the *z*-derivatives of the displacements' Fourier coefficients at 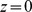, which are unknown *a priori*. The same solution was found independently by three members of our team using Maple (Maplesoft, Waterloo, ON, Canada).

#### 2.6.1 Boundary Conditions on the displacements at *z* = *h*

The three unknown elements of 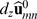 can be determined by imposing the three-dimensional displacements measured at *z* = *h* as boundary conditions (see Fig. 2). For that purpose we determine the Fourier coefficients of the measured displacements, 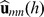, and invert equation 3 particularized at *z* = *h* to arrive at
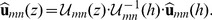
(4)


Explicit analytical expressions for the nine elements in 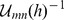 are given in the Supporting Information (File S1). As noted by Butler *et al.* [12], working in the Fourier domain allows us to perform this inversion exactly and without regularization, which can be a delicate issue in methods that work in the physical domain [10]. Analytical differentiation of the resolvant matrix yields explicit formulae for the *z*-derivatives of the displacements,
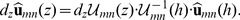
(5)


#### 2.6.2 Determination of the normal and tangential traction stresses on the deformed surface of the substratum

This section employs the solution to the elastostatic equation (1) to determine the Green's function 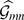 that relates the 3D traction stress vector exerted by the cell on the surface of the gel, 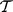, to the measured displacements, 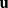. In Fourier space, this relation is given by
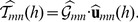
(6)


The stresses on the surface of the substratum are given by by 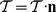, where 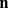 is the unit vector normal to the surface of the substratum, and 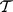 is the three-dimensional stress tensor. Assuming that the deformed surface of the gel is given by 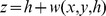, the vector normal to the deformed surface is
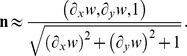



The normal stresses on the deformed substratum are then given respectively by
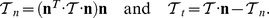



Consistent with the small-deformation approximation [17], the values of 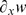 and 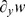 in our experiments are typically small, in the range 0.05–0.1. Thus, 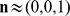, and the normal and tangential stresses can be approximated by 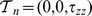 and 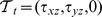, similar to the undeformed conditions. Likewise, the normal and tangential deformation can be expressed as 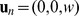 and 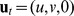.

The stress vector can be obtained from the calculated displacements and their *z*-derivatives by applying Hooke's law in Fourier space,
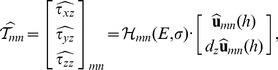
(7)where the 6×3 matrix 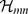, which only depends on the material properties of the substratum (*E* and *σ*) and the wavenumbers of each Fourier mode (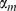, 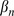), is given in the Supporting Information (File S1). Plugging the solution for 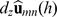 obtained by applying the boundary conditions (equation 5 at *z* = *h*), we obtain the Green's function,
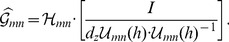
(8)


#### 2.6.3 Determination of the Strain Energy Exerted by the Cells on the Substrate

The strain energy deposited by the cell on the substratum is equal to the mechanical work exerted by the cell and, thus, it can be easily determined using the principle of virtual work [12], [17]. In the 3D case, it is convenient to decompose the strain energy in its normal (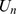) and tangential (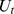) components,
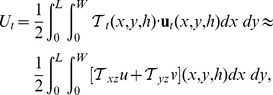
(9)

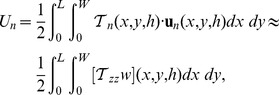
(10)so that 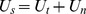 is the total strain energy. These two components can be interpreted as the mechanical work exerted by the tangential and normal traction stresses.

## Results

### 3.1 Application of 3D Fourier Traction Force Microscopy to Migrating Amoeboid Cells

**Figure 3:**
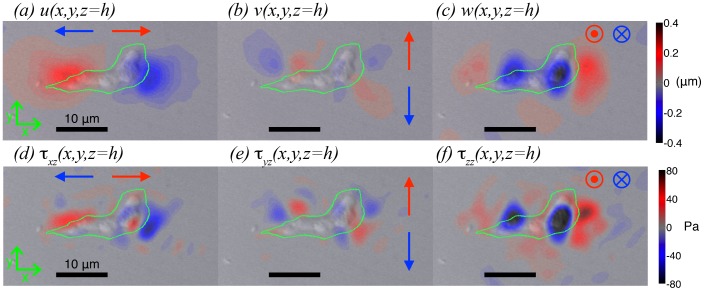
Three-dimensional deformation field and stress field generated by the example cell in Figure 1 on the surface of the substratum. The cell is moving from left to right. The level of deformation/stress is represented by a pseudo-color map according to the color bars at the right hand side of the figure. The green contour indicates the cell outline. The data are overlaid on the DIC image used to identify the cell body (see 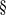 2.4). The black scale bars are 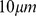 long. *(* ***a*** *)*, Tangential (horizontal) deformation in the direction parallel to cell speed, 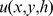. *(* ***b*** *)*, Tangential (horizontal) deformation in the direction perpendicular to cell speed, 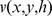. *(* ***c*** *)*, Normal (vertical) deformation, 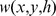. *(* ***d*** *)*, Tangential (horizontal) stress in the direction parallel to cell speed, 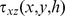. *(e)*, Tangential (horizontal) stress in the direction perpendicular to cell speed, 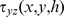. *(f)*, Normal (vertical) stress, 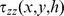. The arrows in panels , , and indicate the directions of positive (red) and negative (blue) deformation/stress. The 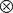 and 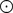 symbols in panels and indicate deformation/stress pointing respectively into the plane (blue, negative) and out of the plane (red, positive).

Consistent with previous 2D studies [13], [22], [23], we found that cells contract the substratum from front and back towards the center of the cell. However, the 3D measurements revealed that the deformation and stresses in the direction perpendicular to the substratum cannot be neglected, as they can be as large as the tangential ones. These observations are illustrated in Figure 3, which displays an example of the deformation and stress fields caused by a migrating *Dictyostelium* cell on the surface of the substratum. The cell in this figure is the same one represented in Figure 1 and is migrating from left to right. As a matter of fact, for the specific cell and instant of time depicted in Figure 3, the peak normal deformation and stresses are even higher than the peak tangential ones. The corresponding normal strain energy, 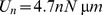, is also greater than the tangential one, 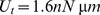 (note that 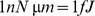).

The 3D measurements also indicate that, during migration, the cells not only tugged the substratum horizontally but also pulled upwards (away from the substratum) along their front and back while pushing downwards right under their center (see Fig. 3*c, f*). These results are in good agreement with previous 3D traction stress measurements [25].

### 3.2 Comparative analysis of 3D and 2D traction force microscopy methods

This section compares the present 3D TFM method with previous 2D TFM methods that assumed the substratum to be infinitely thick and neglected the vertical displacements [10], [12], and with 2D TFM methods that accounted for the finite thickness of the substratum but neglected either the vertical displacements [15] or the vertical traction stresses at *z* = *h* [13]. The comparison is simplified because, under the small deformation-approximation (see 2.6.2), the vector normal to the free surface of the substratum is the same in two and three dimensions. Thus, the *x*–*y* components of the 2D traction stress vector, 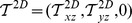, are homologous to those obtained by 3D TFM, where 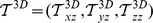. We will thus reduce our analysis to a direct comparison between 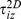 and 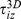 for *i* = *x*,*y*,*z*. Note that in general 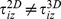 because of the different boundary conditions employed in each case to solve equation 1.

#### 3.2.1 Synthetic-field analysis of 3D and 2D traction force microscopy methods

**Figure 4:**
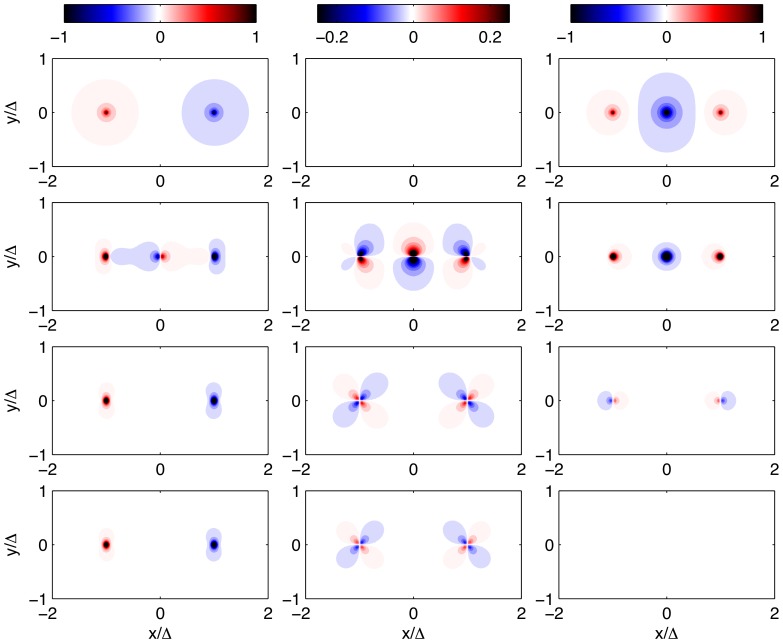
Side-by-side comparison of 3D Fourier TFM versus previous 2D methods [13], [15] for a synthetic deformation field representative of the deformation patterns exerted by migrating amoeboid cells (see Figure 3). The Poisson's ratio is 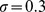 and the substratum thickness, 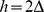, is equal to the length of the “synthetic cell”. The plots in the top row show the synthetic deformation field in the *x* direction (eq. 11, panel *a*), *y* direction (zero, panel *b*) and *z* direction (eq. 13, panel *c*). The second row shows the traction stresses calculated from the displacements in panels (*a*)–(*c*) by 3D Fourier TFM. (*d*), 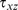; (*e*), 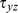; (*f*), 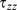. The third row shows the traction stresses calculated from the displacements in panels (*a*)–(*c*) by 2D Fourier TFM under the assumption of zero normal displacements on the substratum's surface (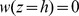 as in ref. [15]). (*g*), 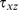; (*h*), 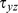; (*i*), 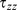. The last row shows the traction stresses calculated from the displacements in panels (*a*)–(*c*) by 2D Fourier TFM under the assumption of zero normal stresses on the substratum's surface (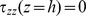 as in ref. [13]). (*j*), 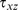; (*k*), 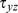; (*l*), 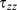.

In order to systematically study the errors in 2D TFM, we calculate the traction stresses generated by a synthetic deformation field applied on the surface of the substratum,
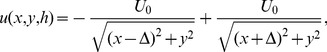
(11)

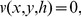
(12)

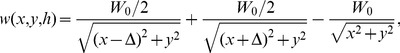
(13)which is plotted in Figures 4(*a*)–(*c*). This synthetic field was chosen because it resembles the deformation pattern caused by migrating amoeboid cells in our experiments (see Figure 3). It consists of three deformation poles whose positions are aligned in the direction parallel to the cell's longitudinal axis (the *x*-axis). The front and back poles are placed at a distance 2Δ to each other. They contract the substratum tangentially towards their midpoint, and pull away from substratum in the direction normal to the free surface. The central pole presses down the substratum in the normal direction. The average deformation created by these three poles is zero in all directions so that static equilibrium is fulfilled.

In order to characterize the dependence of the error on the ratio of tangential to normal deformation, we let *u* and *w* be proportional to two different lenghtscales, *U*_0_ and *W*_0_. Once we set the value of the ratio *W*_0_/*U*_0_, the actual magnitude of *U*_0_ is irrelevant for the purpose of comparing 2D and 3D TFM equations because the elastostatic equation and the constitutive equations of the substratum are linear. The same applies to the magnitude of the substratum's Young modulus. Therefore, we set *U*_0_ = 1 (units length) and *E* = 1 (units force per unit area) for simplicity. The deformation field caused by each pole was chosen to decay as the inverse distance to each pole because this rate of decay is known to result from applying a point force (i.e. a Dirac delta traction stress) at the pole when *h*»Δ [17]. This particular choice allowed us to verify the correctness of our calculations and is immaterial to the comparison of 2D and 3D methods presented below. Figure S1 in the Supporting Information (File S1) shows that the same comparative results are obtained for other choices of the deformation field. The only relevant non-dimensional parameters for this comparison are the ratio Δ/*h*, which can be interpreted as a surrogate for cell length relative to substratum thickness, the Poisson's ratio of the gel, and the ratio of normal to tangential deformation, *W*_0_/*U*_0_. Note that *W*_0_/*U*_0_ is somewhat related to the vertical aspect ratio of the cell; flat cells are geometrically constrained to generate low values of *W*_0_/*U*_0_ while round cells may generate high values of *W*_0_/*U*_0_.

In order to quantify more precisely the accuracy of 2D TFM, and its dependence on the Poisson's ratio, 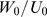 and 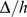, we define the error in the tangential stresses as 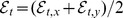, where
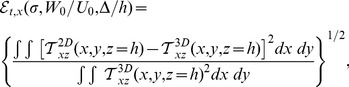
(14)and 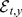 is defined in a similar way. The relative error in the normal stresses is defined as
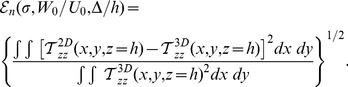
(15)


These errors are normalized so that they are equal to one when 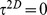 and 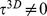.

#### 3.2.2 Influence of the Poisson ratio and cell length on the error of 2D TFM

Based on the evidence that the level of normal deformation is comparable to the level of tangential deformation (see 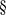 3.1), our analysis will focus first on the dependence of the error on *σ* and 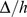 for the case 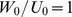. For each pair of values of 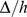 and Poisson's ratio, one can calculate a traction stress map from the boundary conditions specified by the synthetic deformation field in eqs. 11–13 (see Figure 2). Figure 4(*d*)–(*f*) shows the traction stresses obtained by 3-D Fourier TFM for 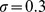 and 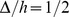, which are representative values of the experimental conditions in TFM experiments [13], [23], [24] The lower panels in that figure display the stresses obtained from the synthetic deformations by 2D TFM methods on finite-thickness substrata [13], [15]. These stresses are obtained by replacing the boundary condition (13) with either 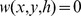 (panels *g*–*i*, ref. [15]), or with 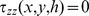 (panels *j*–*l*, ref. [13]).

A visual inspection of Figure 4 provides overall qualitative information about the accuracy of 2D TFM in comparison to the 3D approach introduced in this work. Both 2D methods produce similar results and are able to reproduce the tangential contractile stresses along the *x* direction (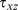) at the front and back poles (Fig. 4*d, g, j*). However, the 2D methods miss the tangential expansive stress caused by the central pole's pushing down into the substratum, which is due to the Poisson's effect. The accuracy of the 2D methods is somewhat worse for the tangential stresses in the *y* direction (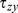, Figure 4*e, h, k*) because these stresses appear exclusively due to the Poisson's effect caused by the deformations prescribed in the other two directions. Finally, and as expected, 2D TFM severely underpredicts the normal traction stresses (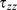, Figure 4*f, i, l*). The 2D methods that impose 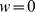 on the surface of the substratum [15] only capture the normal stresses caused by the tangential deformation due to the Poisson's effect. And obviously, the 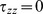 condition [13] leads to zero normal stresses on the surface of the substratum.

**Figure 5:**
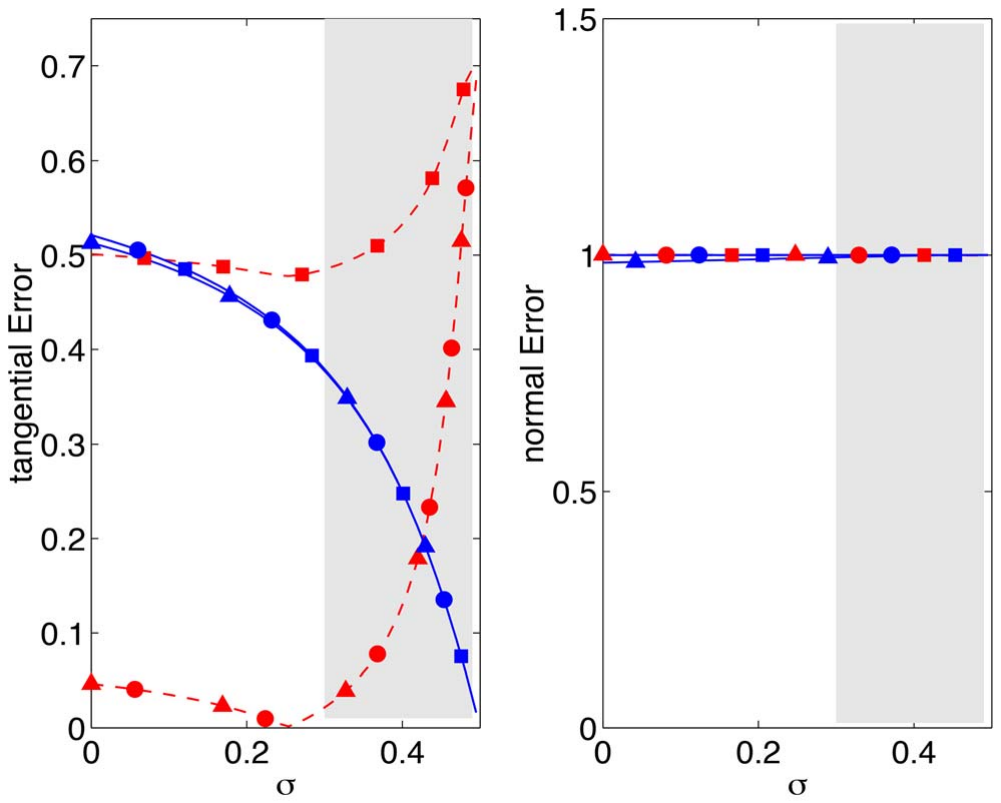
Relative error of 2D TFM methods represented as a function of the Poisson's ratio, *σ*, for two values of the ratio 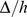
 **.** Red lines and symbols, 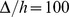; blue lines and symbols, 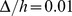.–•–, 2D method with finite *h* and 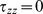 on the surface (ref. [13]);–▴–, 2D method with finite *h* and 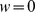 on the surface (ref. [15]);–▪–, Boussinesq solution with infinite *h* (refs. [10], [12]). (*a*), 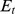; (*b*), 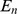. The shaded patch represents the range of values of Poisson's ratio reported for gels customarily employed in TFM [26]–[28].

Figure 5(*a*) displays the tangential errors as a function of the Poisson's ratio for two extreme values in which 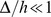 (0.01) and 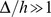 (100). It is worth studying these limits in detail because they contain zones of low 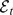, which can be used to guide the design of 2D TFM experiments that are accurate independent of the normal stresses exerted by the cell. The typical range of Poisson's ratios of the gels employed in traction force microscopy, 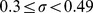 [26]–[28], is indicated by the shaded regions in the plot.

For low values of 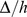 (blue lines in Fig. 5*a*), the length of the cell is much smaller than the thickness of the substratum. In this case, Boussinesq's elastostatic solution for infinitely thick substrata [12] becomes equal to the two finite-thickness solutions [13], [15], which are also approximately equal to each other consistent with Figure 4. Hence, the tangential errors of all three 2D TFM methods are the same for 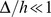. In the range of interest of Poisson's ratios, 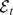 from 2D TFM methods is up to 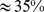 but this error decreases sharply as the Poisson's ratio approaches the incompressible limit, 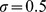. This behavior is explained by recalling that the tangential stresss/strain fields in the Boussinesq solution decouple from the normal ones in that limit (ref. [17], page 25, eq. 8.19). Ideally, it would not be necessary to measure the normal displacements in order to accurately determine the tangential stresses under these conditions (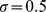, 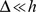). In practice, however, the sharp decrease of 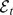 means that this error remains relatively high for values of the Poisson's ratio close to 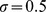. For instance, we obtain that 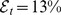 for 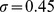.

For high values of 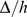 (red lines in Fig. 5*a*), the length of the cell is much larger than the thickness of the substratum. In consequence, Boussinesq's solution yields errors 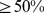 for all values of the Poisson's ratio (Fig. 5). The finite-thickness 2D TFM methods [13], [15] yield relatively low tangential errors as long as the substratum is far from incompressible but their 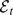 increases abruptly as the Poisson's ratio approaches 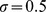. For reference, this error is 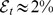 for 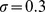 but it becomes 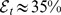 for 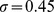. The reason for this behavior can be understood by analyzing the elastostatic equations (1) for very thin substrata. In this limit, the *z*-derivatives are much larger than the *x*,*y*-derivatives and the equations are simplified to
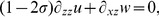
(16)

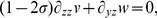
(17)

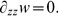
(18)


This simplification shows that, if *σ* is far from 0.5, the second terms in equations (16)–(17) are negligible as they contains *x* and *y* derivatives. In that case, the normal displacements decouple from the tangential ones and it is not necessary to measure the former in order to determine the latter. However, as *σ* approaches 0.5, the factor 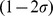 that multiplies the first terms in equations (16)–(17) also becomes small, the *x* and *y* derivatives in those equations cannot be neglected anymore, and the tangential displacements remain coupled to the normal ones.

**Figure 6:**
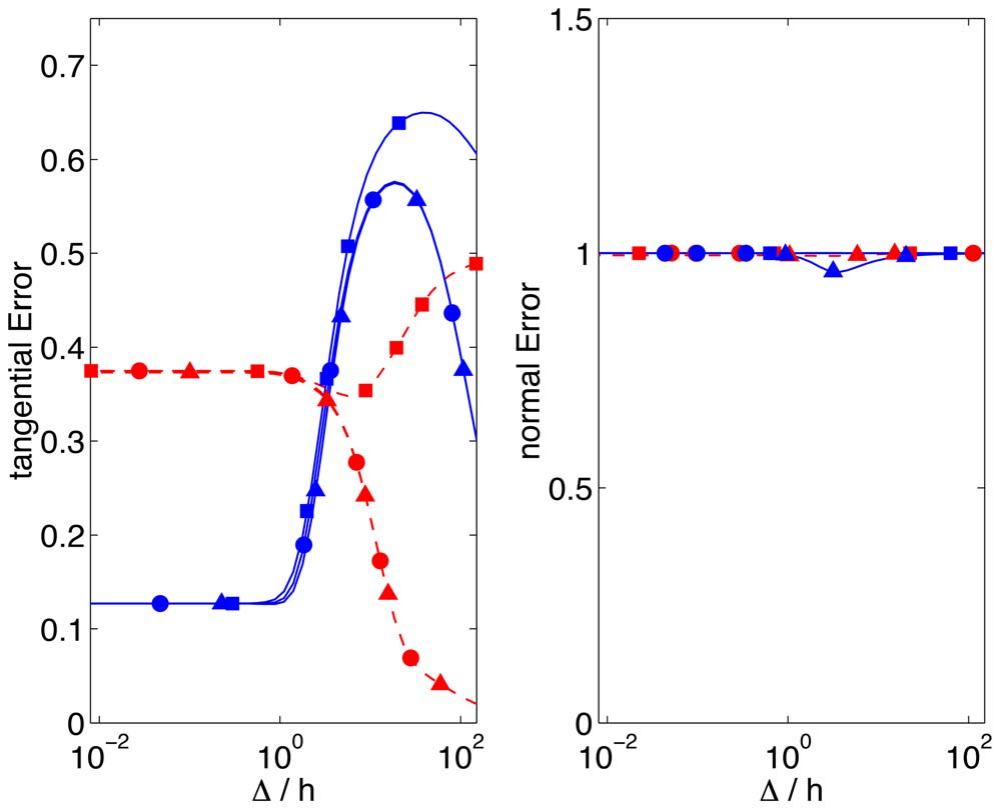
Relative error of 2D TFM represented as a function of 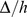 for two values of the Poisson's ratio representative of polyacrylamide gels. Red lines and symbols, 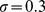; blue lines and symbols, 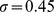.–•–, 2D method with finite *h* and 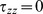 on the surface (ref. [13]);–▴–, 2D method with finite *h* and 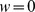 on the surface (ref. [15]);–▪–, Boussinesq solution with infinite *h* (refs. [10], [12]).

Figure 6 is the dual of Figure 5. It illustrates the dependence of 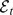 and 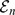 on 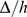 for two values of the Poisson's ratio typical of TFM experiments, namely 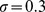 and 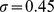 [26]–[28]. For 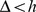, the tangential error of all 2D TFM methods is independent of 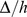 and relatively high (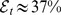 and 13% respectively for 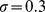 and 0.45, Fig. 6). For 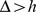, 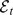 from Boussinesq's 2D method increases steeply with 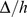, reaching values that exceed 100% for 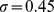. As mentioned above, this large error is due again to the assumption of infinite-thickness substrate made in Boussinesq's solution to the elastostatic equation. The 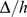-dependence of the tangential error of the finite-thickness 2D TFM methods varies strongly with *σ* for 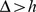, consistent with the results in Figure 5(*a*). For 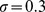, the tangential error decreases monotonically to zero with 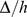, whereas for 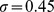, 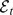 reaches a maximum before decreasing.

**Figure 7:**
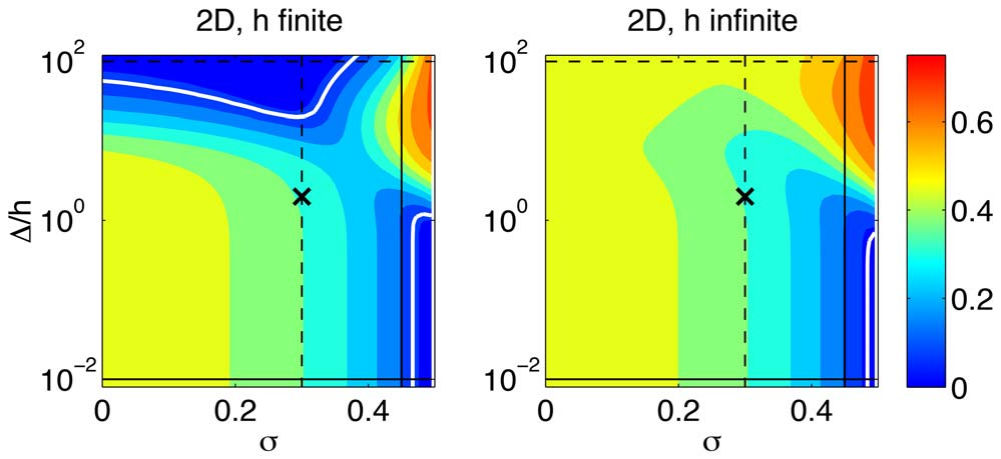
Relative error of 2D TFM represented as a function of the Poisson's ratio, *σ*, and the ratio 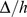. (*a*), 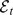 assuming zero normal deformation on the surface of the substratum; (*b*), 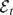 from Boussinesq's solution. The black×marks the combination of *σ* and 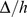 used to calculate the traction stress maps in Figure 4. The horizontal lines mark the values of 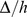 used to plot Figure 5.–––, 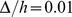;– – –, 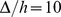. The thick white contours correspond to 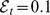. The vertical lines mark the values of *σ* used to plot Figure 6.– – –, 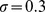;––––, 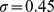.

Figure 7 summarizes the dependence of 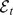 on the Poisson's ratio and 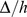 for both finite-thickness 2D methods (panel *a*) and the Boussinesq method (panel *b*). For reference, note that the line plots in Figures 5(*a*) and 6(*a*) are respectively horizontal and vertical 1D cuts of the contour maps in Figure 7. The thick white contours in this figure enclose the regions of the 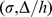 domain where 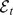 is lower than 10%. Consistent with Figures 5(*a*) and 6(*a*), we find low errors in two regions: one corresponding with small cell size compared to substratum thickness (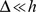) and incompressible gel (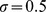), and another region corresponding with large cell size compared to substratum thickness (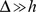) and relatively low Poisson's ratio 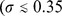). The first low-error region (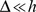, 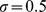) is also obtained for Boussinesq's method but this region turns out to be narrow because 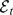 is locally sensitive to small changes in *σ*. This sensitivity leads to significant error values within the range of Poisson's ratios typically encountered in TFM experiments. The second low-error region (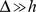, 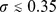) seems to be more robust for the design of TFM experiments because 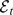 shows a mild local dependence on both the substratum thickness and Poisson's ratio, particularly around 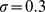. This region is not observed in Boussinesq's method, in which the assumption of infinitely thick substrate is incompatible with Δ being larger than *h*.

For the sake of completeness, the errors of all 2D TFM methods in the normal direction are shown in Figures 5(*b*) and 6(*b*). These errors are 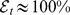 regardless of the boundary condition applied on the gel's surface, and the Poisson's ratio, reflecting the difficulty of determining the normal traction stresses without measuring the normal deformation of the substratum.

#### 3.2.3 Influence of the ratio of normal to horizontal deformation on the error of 2D TFM

Flattened or elongated cells such as epithelial cells or neurons are geometrically constrained and, thus, they are likely to generate predominantly tangential deformations along significant portions of their basal surface. On the other hand, rounder cells such as cancer cells invading into 3D matrices exert predominantly normal deformations [9]. The aim of this section is to characterize the performance of 2D TFM methods under these different scenarios by quantifying their error as a function of the ratio of normal to horizontal deformation (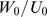 in eqs. 11–13). As noted above, 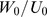 is loosely related to the vertical aspect ratio of the cell, as one can argue that 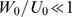 for flat cells and 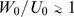 for rounder cells.

**Figure 8:**
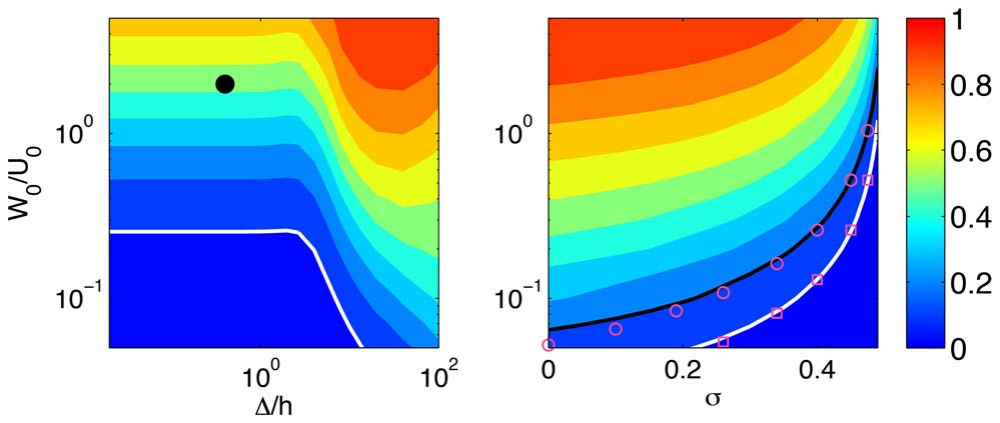
Effect of the ratio of normal to horizontal deformation, 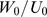 on the error of 2D TFM. (*a*), contour map of 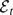 for 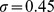, represented as a function of 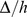 and 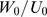. The black circle corresponds to the example cell in Figure 3. The thick white contour corresponds to 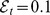. (*b*), contour map of 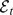 for 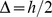, represented as a function of *σ* and 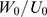. The thick white (black) contours correspond to 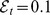 (0.2), and are well approximated by the magenta squares (circles) coming the eq. 19.

Figure 8(*a*) displays the tangential error of 2D TFM with 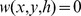 as a function of 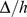 and 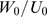. As expected, 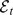 decreases to zero as 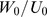 tends to zero and consistent with Figure 7(*a*), this decrease is more gradual in thin substrata. Similar to Figure 7, the isoline 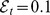 has been outlined with a thick white contour, revealing that 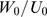 must be lower than 0.25 to achieve a tangential error below 10% for 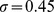. Our analysis suggests that 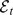 can be relatively high for the values 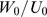 that are often reported in experiments. For instance, the normal deformations are approximately twice larger than the tangential ones for the *Dictyostelium* cell reported in Figure 3, whereas Hur *et al.*'s experiments on endothelial cells are consistent with 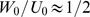 both for single cells and confluent monolayers [5], [6]. In all these cases the tangential error of 2D TFM exceeds 20% according to Figure 8(*a*).

Plotting 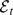 as a function of *σ* and 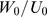 for a fixed value of 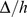 (Figure 8*b*) reveals that, when 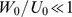, the error of 2D TFM is low and relatively independent of the Poisson ratio. However, this error increases and becomes very sensitive to the Poisson ratio as 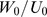 is augmented. Interestingly, we find that the isolines of 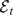 in Figure 8(*b*) are well approximated by the formula
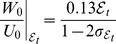
(19)when the error is sufficiently small (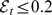). This approximation is found to be uniformly valid for 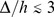, and provides a simple formula to relate 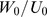 and *σ* for a given level of error. For instance, we establish that 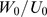 needs to be smaller than 0.26 to keep the error below 10% when the Poisson ratio is 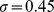. Alternatively, it can be seen that *σ* needs to be higher than 0.487 to keep the error below 10% for 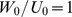.

#### 3.2.4 Spectral analysis of 2D and 3D traction force microscopy methods

**Figure 9:**
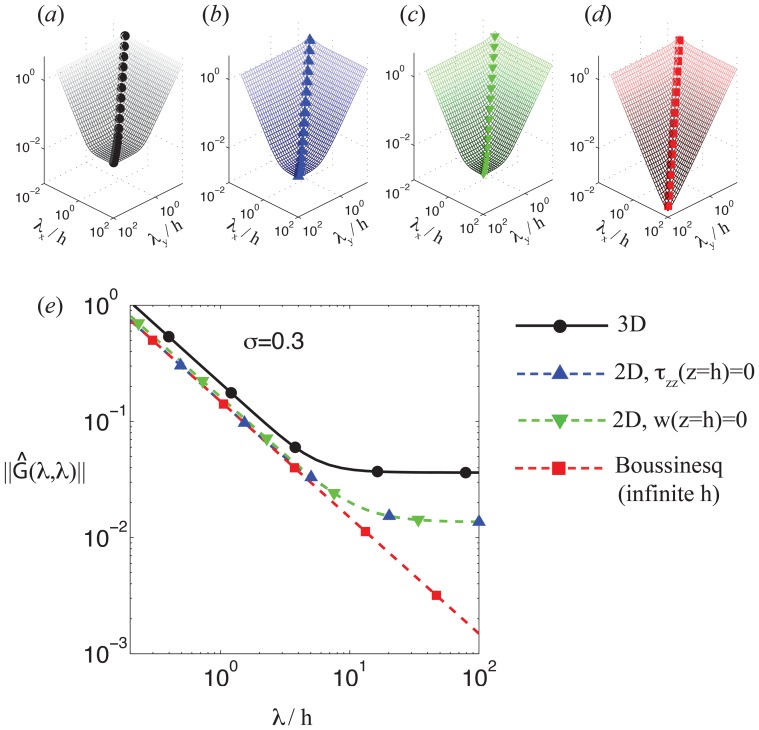
Fröbenius norm of the Green's function used by different TFM methods, 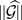 (eq. 20), for 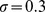. The four panels in the top row (*a–d*) show surface plots of 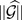 as a function of the horizontal wavelengths of the strain/stress fields 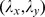.*(* ***a*** *)*, present 3D TFM method;*(* ***b*** *)*, 2D TFM under the assumption of zero normal stresses on the substratum's surface (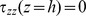 as in ref. [13]);*(* ***c*** *)*, 2D TFM under the assumption of zero normal displacements on the substratum's surface (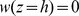 as in ref. [15]);*(* ***d*** *)*, Boussinesq's traction cytometry assuming an infinitely-thick substratum (as in refs. [10], [12]). The symbol curves in these plots indicate the sections of 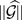 represented in panel (*e*).*(* ***e*** *)*, 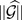 along the line 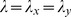 from different traction TFM methods, represented as a function of 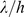.

Fourier analysis of the elastostatic equation (1) provides a general framework to compare different TFM methods that does not rely on any assumption for the shape of the deformation field, and which is complementary to the synthetic-field approach followed in the previous section. This analysis also allows for a general definition of thin substrata as we show below. Figure 9 displays the Fröbenius norm of the Green's functions used in several TFM methods,
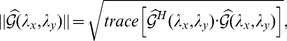
(20)where *^H^* denotes Hermitian transpose. This norm provides a measure of how much a given TFM method amplifies or reduces a harmonic displacement field consisting of wavelengths 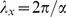 and 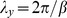 in the *x* and *y* directions. Different to the previous section, where we separately analyze tangential and normal displacements and stresses, the norm employed here provides an overall conservative value that combines the tangential and normal components of 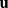 and 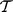. This property is common to most tensorial norms and, therefore, the results from the analysis are independent of the particular choice of norm for the Green's function. In fact, we obtain similar results when using other matrix norms such as the 1-norm, the 2-norm and the ∞-norm.

The wavelengths in Figure 9 are normalized with the thickness of the substratum, *h*, so that high values of 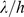 correspond to spatial features of the displacement field that are long compared to the substratum thickness, and vice versa. Overall, we find that 2D TFM methods underestimate 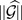, consistent with the results from the previous section. The observed differences are small for low 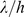 (thick gels). However, the Green's functions of finite-thickness and infinite-thickness TFM methods diverge significantly for 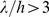, suggesting that the zero-deformation boundary condition imposed at the base of the substratum is felt when the lateral extent of the traction stresses becomes larger than 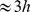. This result provides a natural definition of thin substratum for a given traction stress field; a substratum can be considered as mechanically thin if the dominant wavelength in the deformation field measured on its surface, 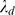, is longer than 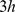. Del Álamo *et al.* [13] showed that 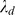 is equal to the cell length in migrating amoeboid cells by measuring the spectral energy density of the deformations exerted by the cells. Although it is possible that 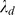 varies with cell type and from single cells to confluent monolayers, the present definition of mechanically thin substratum can be applied to each condition once the deformation field is measured.

This divergence appears to increase as the Poisson's ratio approaches the incompressible limit 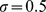, as confirmed by Figure S3 in the Supporting Information (File S1). Note in particular that the Green's functions from finite-thickness 2D TFM methods severely underestimate the 3D Green's function for 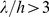. A possible interpretation for this behavior is that normal stresses applied at 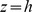 penetrate deeper into the substratum than tangential ones. This hypothesis is further analyzed and confirmed in the next section.

### 3.3 Normal traction stresses reach deeper into the substratum and mechano-sense larger elastic moduli than tangential ones

Del Álamo *et al.* [13] reasoned that the finite-thickness Green's function diverges from Boussinesq's when the 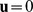 boundary condition imposed at the bottom of the substratum is felt at the substratum's surface. Inspection of Figure 9 reveals that divergence occurs for lower values of *h* in the case of a normal force than for a tangential force, suggesting that cells should be able to feel deeper into the substratum by applying normal forces.

A possible way to quantify this effect is to calculate the apparent elastic moduli of the substratum to tangential and normal traction stresses, 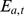 and 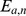 respectively. We define these moduli as the norms of the tangential and normal restrictions of 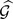 for harmonic deformations of wavenumbers 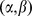 in a substratum of thickness *h* normalized with their value at 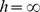. These restricted operators are given by
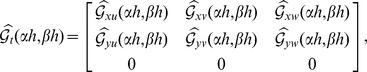
and
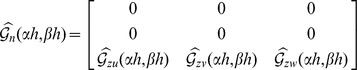
respectively. For simplicity and without loss of generality, we focus on isotropic deformation fields with 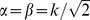 where *k* is the modulus of the wavenumber vector, obtaining
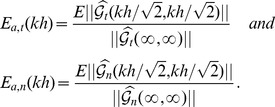
(21)


**Figure 10:**
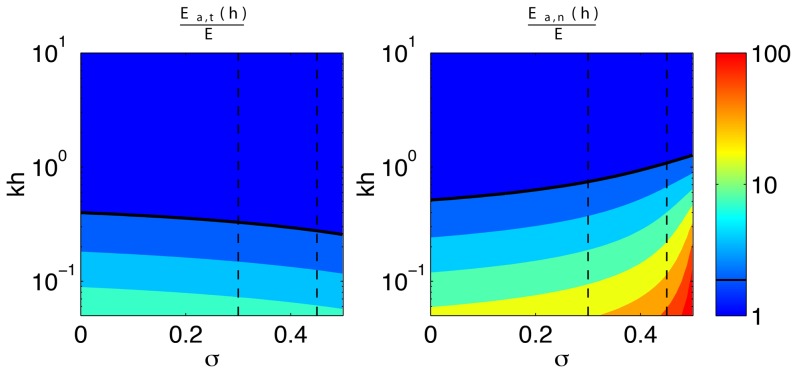
Effect of finite substratum thickness on the substratum stiffness mechanosensed by tangential or normal traction stresses. Panels (*a*) and (*b*) display the apparent elastic modulus of the substratum (eq. 21) in the directions tangential and normal to the surface respectively. The data are plotted as a function of the Poisson's ratio, *σ*, and the substratum thickness *h* normalized with the wavenumber 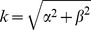 of the strain/stress fields. For simplicity, the 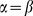 case is represented but similar results are obtained for other combinations of the wavenumbers. The isolines plotted in panels (*a*) and (*b*) are respectively 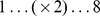, and 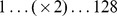. Particularly, the The thick black contour in each panel represents the isoline 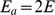 corresponding to a two-fold increase in apparent stiffness. The vertical dashed lines indicate the values of the Poisson's ratio represented in Figure 11, 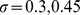.

**Figure 11:**
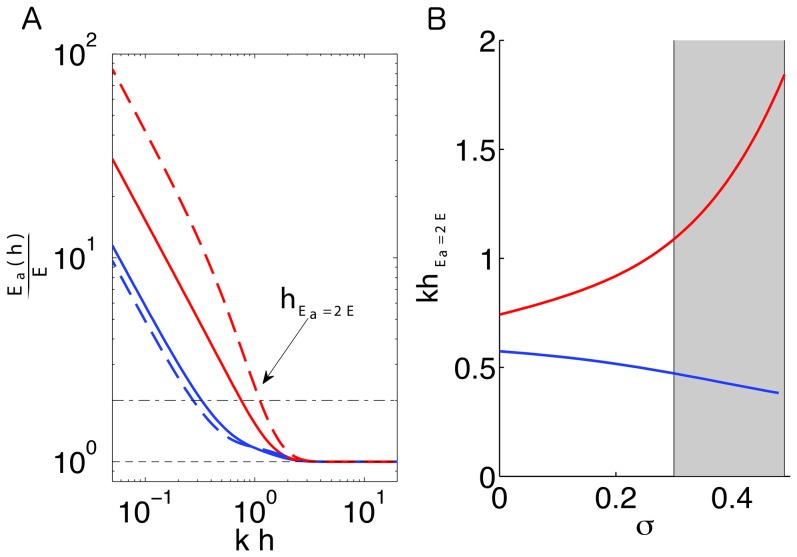
Sensing depth by tangential and normal traction stresses. Panel *(* ***a*** *)* displays the ratio 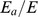 as a function of the substratum thickness *h* normalized with the inverse wavenumber 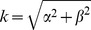 of the strain/stress fields. For simplicity, the 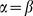 case is represented but similar results are obtained for other combinations of the wavenumbers.–––, normal direction and 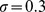;––––, tangential direction and 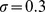;– – –, normal direction and 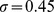;– – –, tangential direction and 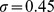. The black horizontal lines indicate the levels 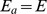 (no increase in apparent elastic modulus,– – –) and 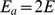 (two-fold increase,–– – ––). Panel *(* ***b*** *)* displays the sensing depth defined as the value of *h* that yields a two-fold increase in apparent elastic modulus compared to 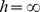. The sensing depth is represented as a function of the Poisson's ratio for tangential and normal traction stresses.–––, normal direction;–––, tangential direction. The shaded patch represents the range of values of Poisson's ratio measured for polymer networks [26]–[28].

Note that 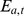 and 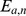 are analogous to the shear and axial elastic moduli of the substratum when measured by applying traction stresses on its free surface. Figures 10(*a*)–(*b*) display two-dimensional contour maps of 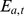 and 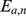 as a function of the Poisson's ratio and the substratum thickness normalized with 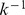, while Figure 11(*a*) shows one-dimensional cuts of these maps for two constant representative values of the Poisson's ratio, 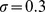 and 0.45. These data demonstrate that, as argued in the introduction, both 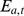 and 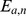 are equal to the bulk Young's modulus of the substratum, *E*, for large values of 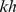. However, the apparent elastic moduli increase steeply as 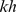 decreases below 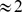, indicating that the substratum becomes effectively stiffer as its thickness decreases. More importantly, the observed increase is significantly more pronounced in 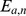 than in 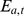, which suggests that the response of thin substrata to normal stresses is stiffer than its response to tangential stresses. We also find that 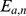 increases strongly with the Poisson's ratio, especially near the incompressible limit, where 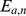 can be >10 times higher than 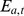. On the other hand, the apparent shear modulus shows a mild decrease with *σ*. These different behaviors can be explained by noting that normal deformations compress the substratum, so it is reasonable to expect that normal forces disturb the substratum more globally and are more affected by substratum compressibility than tangential ones. An increased apparent shear elastic modulus that qualitatively agrees with our predictions was previously found by 2D TFM methods [19]. However, the substantially larger increase in axial apparent modulus predicted by our analysis could not be determined in previous 2D studies.

Altogether, these results confirm that applying normal traction forces at the surface of the substratum provides higher sensitivity to the infinitely rigid bottom than applying tangential traction forces. Or, in other words, that cells may feel deeper into the substratum by normal traction forces. In order to provide a theoretical measure of the sensing depth by tangential and normal forces, we use 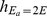 which is the substratum thickness at which the elastic modulus perceived by applying traction forces is twice the Young's modulus. Figure 11(*a*) explains how this sensing depth is calculated and Figure 11(*b*) displays 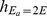 for both tangential and normal forces as a function of the Poisson's ratio. Consistent with the results in Figure 9, we find that the sensing depth is up to 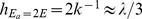, which is equal to 1/3 the lateral extent of the traction stresses exerted by the cell. As expected, normal traction forces lead to larger values of the sensing depth that increase sharply with the Poisson's ratio. On the other hand, tangential forcing leads to lower values of the sensing depth which depend little on the Poisson's ratio. In particular, within the range of values reported for the Poisson's ratio of polyacrylamide gels [26]–[28], 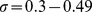 (shaded region in Figure 11*b*), our calculations suggest that normal forces can reach between 2 and 4 times deeper into the substratum than tangential ones.

## Discussion and Conclusion

Adherent cells exert three-dimensional (3D) traction stresses on the extracellular matrix even when cultured on plane surfaces. This study presents a novel traction force microscopy (TFM) algorithm that provides the 3D traction stresses from measurements of the 3D deformation of a thin layer below the surface of the substratum. The new algorithm is based on an exact analytical solution to the equation of mechanical equilibrium for a finite-thickness substratum, using the measured displacements as boundary conditions. The thus obtained solution provides all the elements of the three-dimensional strain and stress tensors everywhere in the substratum, not only the traction stresses in the surface. We have used this new 3D solution to estimate the error of previous two-dimensional (2D) TFM methods, which only provide the in-plane (tangential) components of the traction stress vector and neglect its out-of-plane (normal) component. We have also used this solution to characterize the influence of the finite thickness of the substratum and to evaluate the different apparent elastic moduli that a cell can sense by applying either tangential or normal traction stresses.

Hur *et al.* [5] developed the first TFM method to measure 3D traction stresses generated by cells adhering on plane surfaces. Similar to our study, Hur used the measured 3D deformations at the top of the substratum as boundary conditions to solve the equation of elastic equilibrium. However, instead of finding an exact analytical solution that is compatible with the measured displacements as is done in our study, they employed a finite element solver to determine the 3D traction stresses from the measured deformations. Subsequently, Hur *et al.* [6] extended their method to estimate 3D cell-cell junction forces in cultured cell monolayers. Maskarinec *et al.* [16] proposed an alternate 3D TFM approach that discretizes the whole substratum into a volumetric mesh on which the 3D deformations are measured. All the elements of the 3D strain tensor are then computed on this mesh from the measured deformations, and Hooke's law is directly applied to determine all the elements of the stress tensor on each voxel of the mesh.

The 3D TFM method introduced in this paper has a number of advantages with respect to previously existing methods. Similar to Hur *et al.* [5], the present method only requires acquiring a thin (∼8 µ*m* thickness) *z*-stack composed of few image planes (∼10). This imaging mode allows for a high temporal resolution as the acquisition time of a confocal z-stack is roughly proportional to the number of recorded planes. Imaging in a thin layer also reduces the amount of laser radiation that is imparted on the cells, thereby minimizing phototoxic effects. Furthermore, solving the elastostatic equation (1) instead of directly applying Hooke's law has the advantage of not requiring *z*-derivatives, which can be relatively inaccurate due to the stretch of the point-spread function of the optics in the *z*-direction.

An additional advantage of the method presented here is that it employs an exact analytical solution of the elastostatic equation (1), which allows for computing the traction stresses in virtually zero time from the deformation at 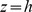. This is made possible by working in the Fourier domain and using Fourier Transform (FFT) routines. The in-house 3D image correlation techniques employed to determine the deformations from the microscopy images also employ FFTs, which further improves the computational efficiency of the present algorithm. The combined features of our method allow us to completeley calculate the traction stress field from a raw microscopy image stack in 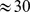 seconds on a laptop computer. In comparison, Maskarinec *et al.* [16] report overall computational times of 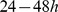 per experiment. Hur *et al.* [5] report computational times of 5 mins for their finite element calculation but this does not include the additional processing time required to measure the deformation field, which is not reported.

Nevertheless, the most important feature of working with an analytical solution is not that it saves us computational time. For the size of the computational problem involved in a TFM experiment, one could always resort to a faster computer, or parallelize the software routines and run them in multiple processors to speed up the computation. The most valuable aspect of an analytical 3D TFM solution is that it offers us a comprehensive tool to analyze the influence of all the parameters involved in the problem. In this work, we have exploited this important feature to characterize the error incurred by the 2D TFM methods in calculating the in-plane traction stresses as a function of cell size, substratum thickness and Poisson's ratio. The aim of this characterization is to establish the range of experimental parameter values within which 2D TFM experiments may still be used to accurately measure the in-plane stresses regardless of the fact that the cell also generates normal traction stresses. By analyzing the error of the 2D methods in a synthetic 3D deformation field that mimics the deformation caused by migrating cells, we have shown that 2D TFM methods that consider the finite thickness of the substratum [13], [15] may perform relatively well when the substratum is exactly incompressible (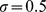) and its thickness is larger than the cell's length. However, the error of 2D TFM is found to be very sensitive to small variations of the Poisson's ratio near the incompressible limit, and to increase considerably when the Poisson's ratio is slightly smaller than 0.5. For instance, we estimate that a typical experiment performed on a 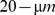-long cell seeded on a 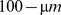-thick substrate of 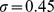 yields errors 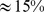 in the tangential traction stresses when the vertical deformations are neglected, and this error is even larger if *σ* is further decreased. We also find a second parametric region resulting in low 2D TFM errors when the cell length is much larger than the thickness of the substratum and the Poisson's ratio is lower than 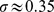. In this second region, the error in the tangential traction stresses shows a mild dependence on both the substratum thickness and Poisson's ratio, especially near 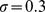, thereby providing a more robust set of experimental conditions than the first region discussed above. It is important to note, nonetheless, that 2D TFM methods that assume an infinitely thick substratum yield errors ≈50% when the cell length is much larger than the substratum thickness.

Given that the traction stresses are proportional to the Young's modulus of the substratum, significant efforts are generally devoted to the characterization of this parameter in TFM experiments. On the other hand, the Poisson's ratio usually receives less attention under the general assumption that this parameter barely influences the traction stresses. The results of this study call for a reexamination of that assumption, as they reveal a strong *σ*-dependence of both the normal traction stresses and the error of the tangential traction stresses. The observed sensitivity to the Poisson's ratio is especially significant for the nearly incompressible gels that are typically used in TFM experiments [26]–[28]. For the above example TFM experiment (a 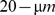-long cell seeded on a 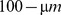-thick substrate of 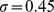), we estimate that a 10% variation in *E* leads to a 10% variation in the traction stresses, whereas a similar error in *σ* (from 0.45 to 0.495) leads to a ≈38% variation in the traction stresses.

It should be noted that, although this study focused on cells adhered onto 2D surfaces, the 3D Green's functions derived here could be applied to determine the traction stresses created by living cells in 3D matrices. In the past, this problem has been tackled using finite element methods [29]. We speculate that working with exact analytical solutions in Fourier space could be advantegeous in terms of convergence and numerical efficiency, as this formulation renders the matrices involved in the problem block-diagonal [12].

**Figure 12:**
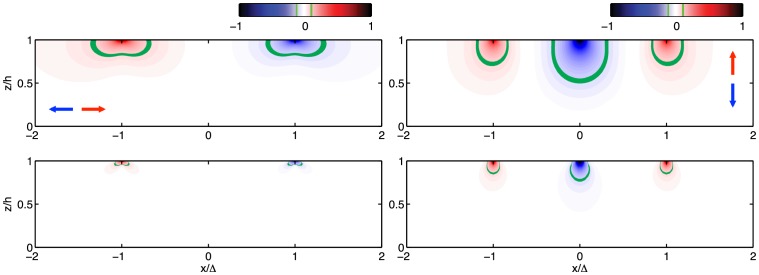
Penetration of tangential and normal deformations and stresses into the substratum. (*a*) Vertical contour map of *u* obtained by applying a unit tangential synthetic deformation field at the free surface of the gel (eqs. 11–13 with 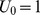 and 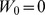), and solving the elastostatic equation for different values of *z*. The deformation is plotted in the normal plane 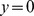 as a function of 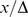 and 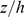. Thus, 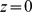 represents the bottom of the gel in contact with the coverslip and 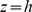 represents the free surface of the gel. (*b*) Same as (*a*) for the normal deformation *w* obtained by applying a unit normal deformation at the gel surface (eqs. 11–13 with 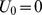 and 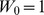). (*c*) Same as (*a*) for 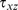. (*d*) Same as (*b*) for 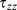. In all panels, the data are normalized between −1 and 1, and the green contour represents the 10% iso-level.

Adherent cells have been reported to sense the mechanical properties (Young's modulus) of the extracellular matrix by applying traction stresses to it [2], [3]. It is recognized that this sensing process can be affected by the contact of rigid elements with the extracellular matrix (glass at the bottom of substratum in an *in vitro* culture or bone *in vivo*, [30]), leading the cell to sense apparent stiffness values that exceed the bulk properties of the ECM. We defined the apparent elastic modulus of a finite-thickness gel by comparing the stresses generated by applying displacements at its surface with those generated in a gel of infinite thickness. We examined the response of the gel separately for normal and tangential surface displacements, which allowed us to obtain both normal and tangential apparent moduli. Previous 2D studies had only studied the tangential response [18], [19]. Our analysis indicates that the apparent Young modulus of the substratum is affected by the glass coverslip when the dominant wavelength in the deformation field, 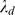, is longer than 3 *h*. This result is in general agreement with the 2D results of Lin *et al.* [19]. However, our three-dimensional analysis indicates that cells can feel between 2 and 4 times deeper into the substratum by generating normal traction forces compared to when they only generate tangential stresses. We have argued that this difference is due to the axial deformation and stress generated by normal traction stresses, which penetrate deeper into the substratum than the shear deformation generated by tangential traction stresses. A clear example of this effect is shown in Figure 12. Our analysis also reveals that the apparent stiffness sensed by to normal traction stresses deformations is considerably higher than the stiffness sensed by to tangential ones. In particular, in the range of Poisson's ratios of the gels that are representative of the extracellular matrix or that are employed to manufacture substrata for *in vitro* TFM experiments [26]–[28], the normal apparent elastic modulus can be >10 times higher than the tangential one. The predicted large disparity is interesting because it could allow investigators to modulate the cell's response to the mechanical properties of the extracellular matrix by altering the ratio between tangential and normal traction stresses. The latter could in turn be controlled via the cell's aspect ratio by micropatterning the substratum [31].

Existing theoretical and experimental studies have suggested that cells mechanosense on length scales similar to that of focal adhesions instead of on cell length [18], [30]. It may be possible to reconcile this evidence with our theoretical study and the experimental data from Lin *et al.* [19] by considering that although 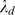 was found to be equal to cell length for *Dictyostelium* cells [13], this dominant wavelength can in principle vary with cell type and other parameters such as cell density (e.g. confluent versus non confluent). Furthermore, our model tacitly assumes that cells mechanosense by sampling substratum deformation but recent data suggests that cells may sample stresses, especially on stiffer substrata [32]. Note that, because stresses come from spatial derivatives of deformations, their dominant wavelength and thus their associated sensing lengthscale is smaller than 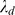.

A potential limitation of the present approach is that, similar to most existing TFM methods, we assume homogeneous, isotropic, linearly elastic mechanical properties for the substratum. While these assumptions facilitate the calculation of an analytical solution to the elastostatic equation, they are not representative of a number of conditions that warrant experimental access to cellular traction stresses, such as in durotactic cell migration on substrata with prescribed stiffness gradients [33]. In those situations, the present Fourier approach can be generalized using regular perturbation expansions [34] to obtain new analytical solutions for the traction stresses that are valid for substrata with slowly varying properties [22]. In conclusion, the 3D TFM method introduced in this paper enables the theoretical and experimental study of new aspects of cell physiology in more realistic microenvironments.

## Supporting Information

File S1
**Supporting Information.** Figure S1. Side by side comparison of 3D Fourier TFM versus previous 2D methods [13], [15] for a synthetic deformation field representative of the deformation pattern exerted by migrating amoeboid cells. The Poisson's ratio is 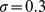 and the substratum thickness, 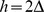, is equal to the length of the “synthetic cell”. The plots in the top row show the synthetic deformation field in the *x* direction (eq. 11, panel *a*), *y* direction (zero, panel *b*) and *z* direction (eq. 13, panel *c*). The second row shows the traction stresses calculated from the displacements in panels (*a*)–(*c*) by 3D Fourier TFM. (*d*), 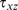; (*e*), 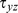; (*f*), 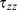. The third row shows the traction stresses calculated from the displacements in panels (*a*)–(*c*) by 2D Fourier TFM under the assumption of zero normal displacements on the substratum's surface (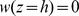 as in ref. [15]). (*g*), 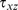; (*h*), 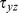; (*i*), 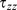. The last row shows the traction stresses calculated from the displacements in panels (*a*)–(*c*) by 2D Fourier TFM under the assumption of zero normal stresses on the substratum's surface (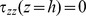 as in ref. [13]). (*j*), 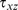; (*k*), 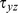; (*l*), 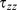. Figure S2. Example of the three-dimensional cross-correlation of fluorescence intensity, 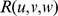, for a pair of interrogation boxes of size 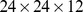 in the *x*, *y* and *z* directions. The three-dimensional location of the peak of the cross-correlation yields the relative displacement between the two interrogation boxes. The signal-to-noise ratio in this example, 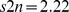, is determined by the ratio of the maximum value of the cross-correlation (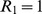) to the second highest local maximum (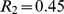). (*a*), Contour map of a two-dimensional section of the cross-correlation for zero displacement in the *z* direction, 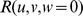. (*b*), Contour map of a two-dimensional section of the cross-correlation for zero displacement in *y* direction, 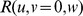. The insets in both panels are height maps of each two-dimensional section of 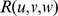. Figure S3. Fröbenius norm of the Green's function used by different TFM methods, 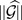 (eq. 20), for a value of the Poisson's ratio 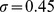. The four panels in the top row (*a–d*) show surface plots of 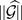 as a function of the horizontal wavelengths of the deformation field 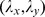.*(* ***a*** *)*, present 3D TFM method;*(* ***b*** *)*, 2D TFM under the assumption of zero normal stresses on the substratum's surface (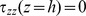 as in ref. [13]);*(* ***c*** *)*, 2D TFM under the assumption of zero normal displacements on the substratum's surface (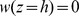 as in ref. [15]);*(* ***d*** *)*, Boussinesq's traction cytrometry assuming infinitely-thick substratum (as in refs. [10], [12]). The symbol curves in these plots indicate the sections of 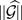 represented in panel (*e*).*(* ***e*** *)*, 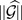 along the line 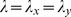 from different TFM methods, represented as a function of 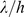.(PDF)
