# Supplementary material for: Three-Dimensional Quantification of Cellular Traction Forces and Mechanosensing of Thin Substrata by Fourier Traction Force Microscopy
Source: PLoS One. 2013 Sep 4;8(9):e69850. doi: 10.1371/journal.pone.0069850 (PMC3762859; doi:10.1371/journal.pone.0069850)
Supplement: File S1 — Supporting Information. Figure S1. Side by side comparison of 3D Fourier TFM versus previous 2D methods [13], [15] for a synthetic deformation field representative of the deformation pattern exerted by migrating amoeboid cells. The Poisson's ratio is and the substratum thickness, , is equal to the length of the “synthetic cell”. The plots in the top row show the synthetic deformation field in the x direction (eq. 11, panel a), y direction (zero, panel b) and z direction (eq. 13, panel c). The second row shows the traction stresses calculated from the displacements in panels (a)–(c) by 3D Fourier TFM. (d), ; (e), ; (f), . The third row shows the traction stresses calculated from the displacements in panels (a)–(c) by 2D Fourier TFM under the assumption of zero normal displacements on the substratum's surface ( as in ref. [15]). (g), ; (h), ; (i), . The last row shows the traction stresses calculated from the displacements in panels (a)–(c) by 2D Fourier TFM under the assumption of zero normal stresses on the substratum's surface ( as in ref. [13]). (j), ; (k), ; (l), . Figure S2. Example of the three-dimensional cross-correlation of fluorescence intensity, , for a pair of interrogation boxes of size in the x, y and z directions. The three-dimensional location of the peak of the cross-correlation yields the relative displacement between the two interrogation boxes. The signal-to-noise ratio in this example, , is determined by the ratio of the maximum value of the cross-correlation () to the second highest local maximum (). (a), Contour map of a two-dimensional section of the cross-correlation for zero displacement in the z direction, . (b), Contour map of a two-dimensional section of the cross-correlation for zero displacement in y direction, . The insets in both panels are height maps of each two-dimensional section of . Figure S3. Fröbenius norm of the Green's function used by different TFM methods, (eq. 20), for a value of the Poisson's ratio . The four panels in t [file pone.0069850.s001.pdf]

Supporting Information

This appendix provides the explicit mathematical expressions to be plugged in the equations presented in §2.6 to determine the traction stresses. These formulae should be preferred to those in the Supporting Information of del Álamo *et al.* [13]. The latter were particularized to two-dimensional boundary conditions and contained a number of typographical errors that have been corrected here. The resolvant matrix of the Fourier transform of the elastostatic equation is

$$\mathbf{U}(\alpha,\beta,z) = \begin{bmatrix} \frac{\alpha^2 z\cosh(k\,z)}{4k^2(1-\sigma)} + \frac{[4(1-\sigma)k^2-\alpha^2]\sinh(k\,z)}{4k^3(1-\sigma)} \\ \frac{\alpha\,\beta\,z\cosh(k\,z)}{4k^2(1-\sigma)} - \frac{\alpha\,\beta\,\sinh(k\,z)}{4k^3(1-\sigma)} \\ \frac{-i\sinh(k\,z)z\alpha}{4k(1-\sigma)} \end{bmatrix} \tag{22}$$

$$\mathbf{V}(\alpha,\beta,z) = \begin{bmatrix} \frac{\alpha\,\beta\,z\cosh(k\,z)}{4k^2(1-\sigma)} - \frac{\alpha\,\beta\,\sinh(k\,z)}{4k^3(1-\sigma)} \\ \frac{\beta^2 z\cosh(k\,z)}{4k^2(1-\sigma)} + \frac{[4(1-\sigma)k^2-\beta^2]\sinh(k\,z)}{4k^3(1-\sigma)} \\ \frac{-i\sinh(k\,z)z\beta}{4k(1-\sigma)} \end{bmatrix} \tag{23}$$

$$\mathbf{W}(\alpha,\beta,z) = \begin{bmatrix} \frac{-i\alpha\,z\sinh(k\,z)}{2k\,(1-2\,\sigma)} \\ \frac{-i\beta\,z\sinh(k\,z)}{2k\,(1-2\,\sigma)} \\ \frac{-z\cosh(k\,z)}{2(1-2\,\sigma)} + \frac{(3-4\,\sigma)\sinh(k\,z)}{2k\,(1-2\,\sigma)} \end{bmatrix}. \tag{24}$$

The inverse of the resolvant matrix particularized at the surface of the substratum is given by

$$\left[\ \mathbf{U}_{mn}(h)\ \middle|\ \mathbf{V}_{mn}(h)\ \middle|\ \mathbf{W}_{mn}(h)\ \right]^{-1} = \begin{bmatrix} C_{1u} & C_{1v} & C_{1w} \\ C_{2u} & C_{2v} & C_{2w} \\ C_{3u} & C_{3v} & C_{3w} \end{bmatrix}, \tag{25}$$

where

$$C_{1u} = \frac{\left(-4\,\beta^2h^2+2\,(-3+4\,\sigma)^2(\cosh(2\,k\,h)-1)\right)k^5+8\,\alpha^2h\,(-1+\sigma)\sinh(2\,k\,h)k^4-2\,\alpha^2(\cosh(2\,k\,h)-1)(-3+4\,\sigma)k^3}{k^4\left(4\,h^2k^2+3\,(-3+4\,\sigma)^2\right)\sinh(k\,h)-(-3+4\,\sigma)^2k^4\sinh(3\,k\,h)},$$

$$C_{1v} = \frac{4\,\alpha\,k^5h^2\beta+8\,\alpha\,\beta\,h\,(-1+\sigma)\sinh(2\,k\,h)k^4+(-2\,\alpha\,\beta\,(-3+4\,\sigma)\cosh(2\,k\,h)+2\,\alpha\,\beta\,(-3+4\,\sigma))k^3}{k^4\left(4\,h^2k^2+3\,(-3+4\,\sigma)^2\right)\sinh(k\,h)-(-3+4\,\sigma)^2k^4\sinh(3\,k\,h)},$$

$$C_{1w} = \frac{-8\,ik^5\alpha\,(-1+\sigma)h\,(\cosh(2\,k\,h)-1)}{k^4\left(4\,h^2k^2+3\,(-3+4\,\sigma)^2\right)\sinh(k\,h)-(-3+4\,\sigma)^2k^4\sinh(3\,k\,h)},$$

$$C_{2u}(\alpha,\beta) = C_{1v}(\beta,\alpha),$$

$$C_{2v}(\alpha,\beta) = C_{1u}(\beta,\alpha),$$

$$C_{2w}(\alpha,\beta) = C_{1w}(\beta,\alpha),$$

$$C_{3u}(\alpha,\beta) = \frac{-4\,i\alpha\,(-1+2\,\sigma)h\,\sinh(k\,h)k^3}{(-3+4\,\sigma)^2k^2\cosh(2\,k\,h)-2\,h^2k^4-(-3+4\,\sigma)^2k^2},$$

$$C_{3v}(\alpha,\beta) = C_{3u}(\beta,\alpha),$$

$$C_{3w}\frac{-4\,k^4 h\,(-1+2\,\sigma)\cosh(k\,h)+4\,\sinh(k\,h)(-1+2\,\sigma)(-3+4\,\sigma)k^3}{(-3+4\,\sigma)^2 k^2\cosh(2\,k\,h)-2\,h^2k^4-(-3+4\,\sigma)^2k^2}.$$

Finally, the linear operator that defines Hooke's law in Fourier space can be written as

$$\mathcal{H} = \frac{E}{2(1+\sigma)}\begin{bmatrix} 0 & 0 & i\alpha_m & 1 & 0 & 0 \\ 0 & 0 & i\beta_n & 0 & 1 & 0 \\ \frac{2i\alpha_m\sigma}{(1-2\sigma)} & \frac{2i\beta_n\sigma}{(1-2\sigma)} & 0 & 0 & 0 & \frac{2(1-\sigma)}{(1-2\sigma)} \end{bmatrix}. \tag{26}$$

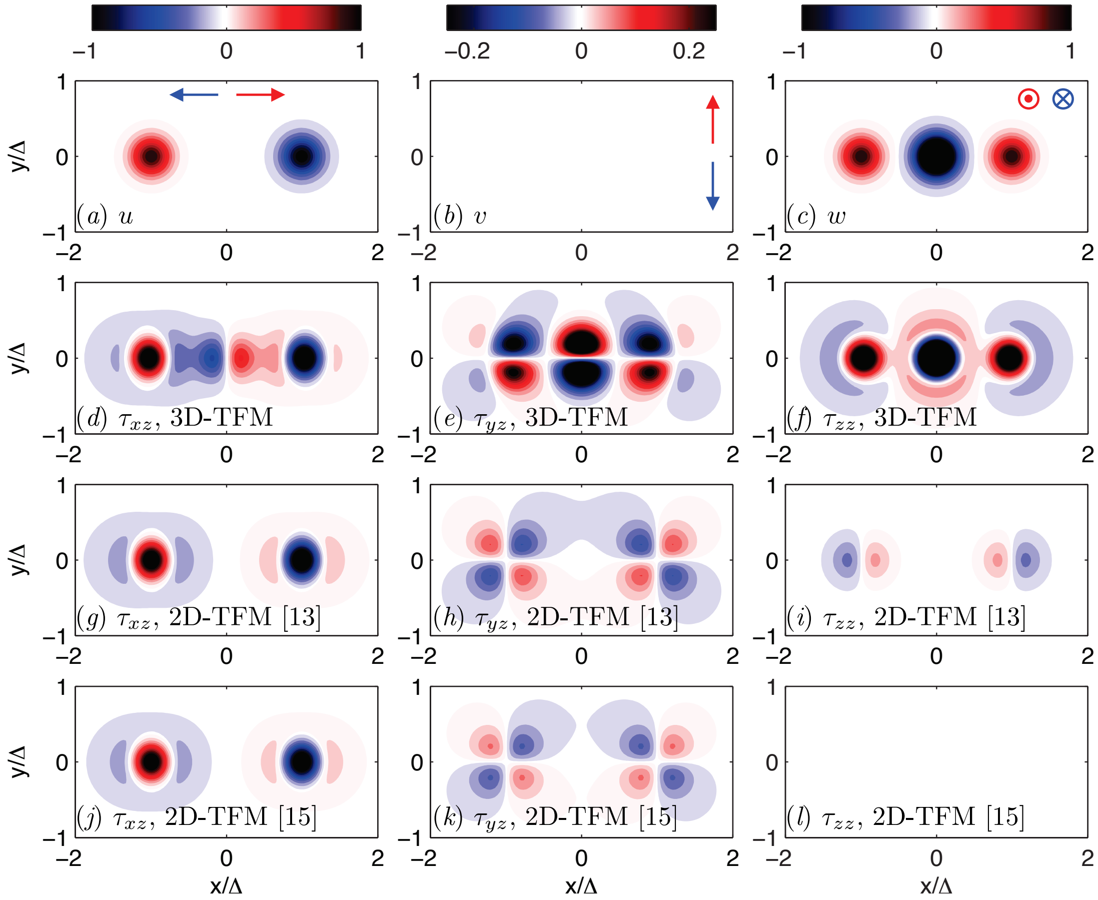

Figure S1. Side by side comparison of 3D Fourier TFM versus previous 2D methods [13, 15] for a synthetic deformation field representative of the deformation pattern exerted by migrating amoeboid cells. The Poisson's ratio is $\sigma = 0.3$ and the substratum thickness, $h = 2\Delta$, is equal to the length of the "synthetic cell". The plots in the top row show the synthetic deformation field in the x direction (eq. 11, panel a), y direction (zero, panel b) and z direction (eq. 13, panel c). The second row shows the traction stresses calculated from the displacements in panels (a)-(c) by 3D Fourier TFM. (d), τ_{xz}; (e), τ_{yz}; (f), τ_{zz}. The third row shows the traction stresses calculated from the displacements in panels (a)-(c) by 2D Fourier TFM under the assumption of zero normal displacements on the substratum's surface (i.e. $w(z = h) = 0$ as in ref. [15]). (g), τ_{xz}; (h), τ_{yz}; (i), τ_{zz}. The last row shows the traction stresses calculated from the displacements in panels (a)-(c) by 2D Fourier TFM under the assumption of zero normal stresses on the substratum's surface (i.e. $\tau_{zz}(z = h) = 0$ as in ref. [13]). (j), τ_{xz}; (k), τ_{yz}; (l), τ_{zz}.

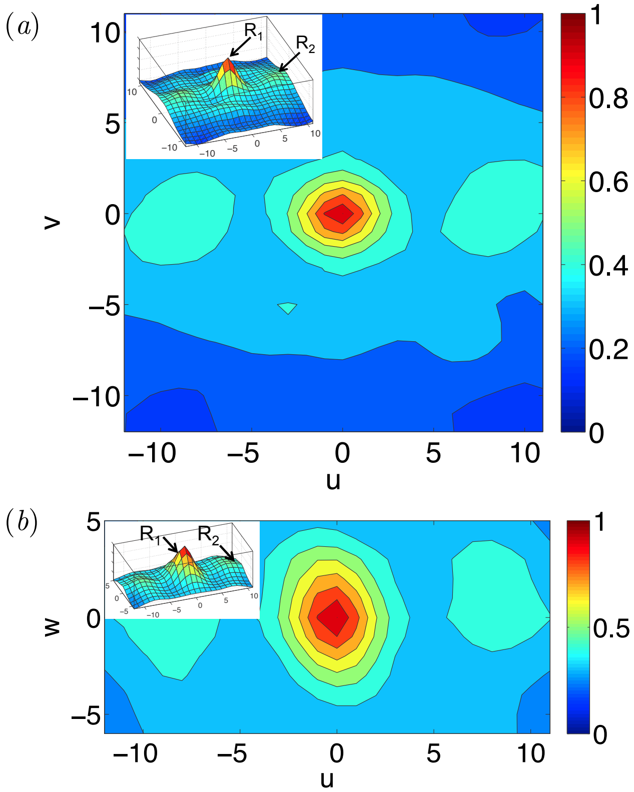

Figure S2. Example of the three-dimensional cross-correlation of fluorescence intensity, $R(u, v, w)$, for a pair of interrogation boxes of size $24 \times 24 \times 12$ in the x, y and z directions. The three-dimensional location of the peak of the cross-correlation yields the relative displacement between the two interrogation boxes. The signal-to-noise ratio in this example, $s2n = 2.22$, is determined by the ratio of the maximum value of the cross-correlation ($R_1 = 1$) to the second highest local maximum ($R_2 = 0.45$). (a), Contour map of a two-dimensional section of the cross-correlation for zero displacement in the z direction, $R(u, v, w = 0)$. (b), Contour map of a two-dimensional section of the cross-correlation for zero displacement in y direction, $R(u, v = 0, w)$. The insets in both panels are height maps of each two-dimensional section of $R(u, v, w)$.

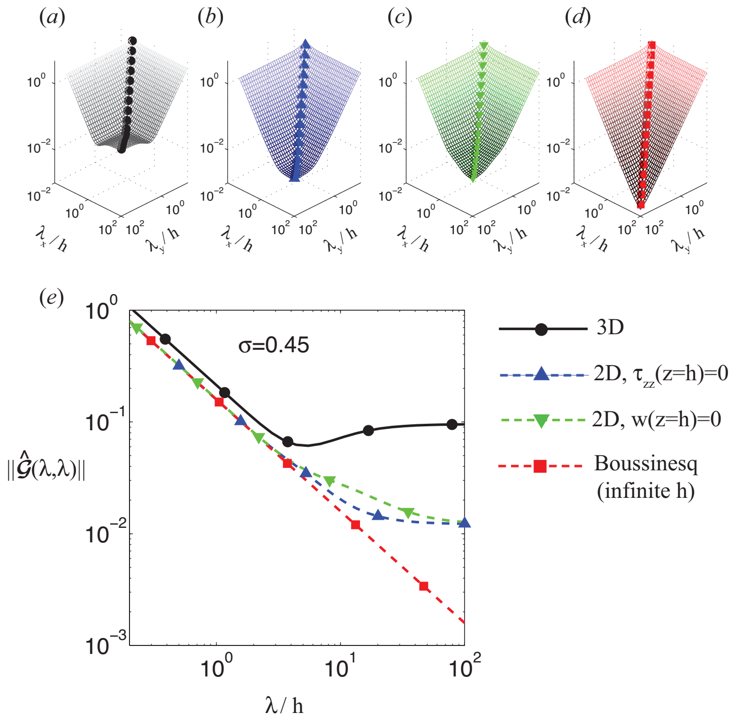

Figure S3. Fröbenius norm of the Green's function used by different TFM methods, $||\widehat{\mathcal{G}}||$ (eq. 20), for a value of the Poisson's ratio $\sigma = 0.45$. The four panels in the top row (a–d) show surface plots of $||\widehat{\mathcal{G}}||$ as a function of the horizontal wavelengths of the deformation field (λ_x, λ_y). ***(a)***, present 3D TFM method; ***(b)***, 2D TFM under the assumption of zero normal stresses on the substratum's surface (i.e. $\tau_{zz}(z = h) = 0$ as in ref. [13]); ***(c)***, 2D TFM under the assumption of zero normal displacements on the substratum's surface (i.e. $w(z = h) = 0$ as in ref. [15]); ***(d)***, Boussinesq's traction cytrometry assuming infinitely-thick substratum (as in refs. [10, 12]). The symbol curves in these plots indicate the sections of $||\widehat{\mathcal{G}}||$ represented in panel (e). ***(e)***, $||\widehat{\mathcal{G}}||$ along the line $\lambda = \lambda_x = \lambda_y$ from different TFM methods, represented as a function of λ/h.
